# Is organic chemistry science – and does this question make any sense at all?

**DOI:** 10.3762/bjoc.11.100

**Published:** 2015-05-27

**Authors:** Andreas Kirschning, Thomas A C Reydon

**Affiliations:** 1 Institute of Organic Chemistry and Center of Biomolecular Drug Research (BMWZ), Leibniz Universität Hannover, Schneiderberg 1b, D-30167 Hannover, Germany; 2 Institute of Philosophy and Centre for Ethics and Law in the Life Sciences (CELLS), Leibniz Universität Hannover, Im Moore 21, D-30167 Hannover, Germany

One could say that we live in the molecular age. The biological and biomedical sciences, the geosciences, medicine and psychology, among others, have all been moving towards a molecular description and understanding of the phenomena they study. In this respect chemistry, and in particular major parts of organic chemistry, play a key role in understanding the living world, as it was here that the foundations of molecular thinking were established.

Despite its huge intellectual and practical impact on other scientific disciplines chemistry is hardly a topic of interest in other academic arenas. It is even largely neglected by philosophers who professionally study the sciences. About a century ago, for example, philosophers vividly debated the natural sciences, fostered by the golden age of theoretical physics. Among the most visible protagonists were the representatives of the Vienna and Berlin Circles, such as Moritz Schlick (1882–1936), Rudolf Carnap (1891–1970), and Hans Reichenbach (1891–1953), as well as Karl Popper (1902–1994). Despite the fact that these times of philosophical discussion were also golden ones for chemistry and its largest field, organic chemistry, chemistry was hardly addressed in these debates [1–2]. To some extent this had to do with the focus of these philosophers on physics as the paradigmatic example of science, i.e., a role model for what science is (for example: thoroughly mathematical and centered on laws of nature). In addition, an important consideration was that chemistry could perhaps be reduced to physics, and chemical phenomena could completely be described in terms of quantum mechanics. On such a view, chemistry would at best be a non-autonomous, applied science that depends for its explanatory content on the laws of more fundamental sciences (a view that is also voiced by some contemporary philosophers with respect to biology, see [3]).

Immanuel Kant (1724–1804), the great German philosopher, too, considered chemistry not to be a proper science, albeit for different reasons. For Kant, the laws of chemistry were “merely” empirical and could not aspire to the status of necessary truths. As Kant argued in his *Metaphysical Foundations of Natural Science* (1786), only disciplines that have a foundation in mathematics and therefore are able to formulate necessary truths on the basis of a priori principles should be counted as proper sciences. Mechanics, for example, was a proper science. Chemistry, in contrast, for Kant was improperly called science, that is, he considered it to be not a science at all but rather a “systematic art” [4]. Note, though, that the qualification of chemistry as a systematic art was not meant as a disqualifier: Systematic arts for Kant were experimental doctrines, that is, the empirical investigation of natural phenomena guided by reason “where we begin by the observable large-scale properties of matter and then attempt to determine its internal structure “from the outside in”” [5]. While this is what today we would call empirical science [6] or applied or practical science [2], the important point is that such investigations do not yield (certain, necessary, a priori) laws of nature, but “merely” empirical descriptions of the phenomena that, according to Kant, to constitute scientific knowledge must be embedded in the system of a priori laws of nature that the proper sciences could yield [2,6].

In practice it may be of little importance whether philosophers regard chemistry to be a “proper” science or something else, such as an applied science or a domain of technology. Still, the character of chemistry plays an important role in determining how chemistry is, and could be, related to other disciplines. Linus Pauling (1901–1994), for example, argued that chemistry could play an integrative role in the domain of science and technology, as chemistry was more general than other disciplines and studied a much wider area of phenomena. As Pauling stressed: “Physicists in general tend to restrict themselves to the small part of the physical world with which they deal, and to leave out of their studies all such features as the structure, and properties of substances in relation to their chemical composition, and the reactions that change one substance into another” [7]. The integrative role that Pauling envisaged for chemistry allocates the field a central and fundamental place among the sciences, which seems difficult to combine with a status of chemistry as applied science or technology. In the philosophy of science the question what kind of enterprise chemistry is, is increasingly coming into focus these days, in part due to the fact that over the past one or two decades the philosophy of chemistry has increasingly established itself as a clearly visible area of investigation [1–2,8]. In addition, this interest is linked to the rapidly emerging molecular bio- and life sciences and their impact on our societies and on the individual. The question what is “proper” science now is also debated in new areas of the biosciences, such as synthetic biology, of which it is claimed that it represents the transformation of biology to technology or to engineering.

So, can chemistry and in particular organic chemistry be thought of as a “proper” science (where the notion of “proper science” still needs to be clarified), or should it be seen as a different sort of discipline, such as an applied science or a technological discipline? We will address this question from a number of perspectives connected to traditional lines of discussion in the philosophy of science and technology. Our aim is to highlight some perspectives from which the issue could nowadays be approached and to bring the issue to the attention of the community of chemists.

First, there is the question what science is, i.e., how science is best characterized. This may seem easy enough to answer: We usually recognize science when we see it. Upon a closer look, however, the question poses curiously difficult problems. Philosophers of science have traditionally tried to answer it by formulating a so-called demarcation criterion that would allow us to distinguish between “good” or “proper” science and non-scientific areas of work, such as pseudo-sciences (fields that pretend to be scientific but in fact are not; think of Intelligent Design) and non-sciences (fields that do not claim to be sciences in any strict sense while still being legitimate areas of work; think of the humanities, but also of the engineering disciplines). The question whether a particular field of work is a science or perhaps rather a technological discipline can thus be conceived of as asking for the application of a demarcation criterion that distinguishes the sciences from other academic fields, including technology. Would this be a promising endeavor?

A famous example of a demarcation criterion is Karl Popper’s notion of falsifiability, according to which those areas of work are scientific that produce statements that can be tested and refuted [9]. Popper’s thoughts on science were strongly influenced by the theoretical developments in physics in the early 20th century, especially the rise of general relativity theory and quantum physics. These new theoretical proposals could only survive if well-designed experiments would not falsify them. Even though chemistry is more like an applied science and often relies on models rather than on pure theories, one can envision fundamental theories that would fall under Popper’s narrow definition that can only or principally be falsified by organic chemists. Some examples of topics in which falsification could play a role located at the interface to the biosciences are:

1. the origin of organic molecules on earth and what is called prebiotic chemistry [10–11];
2. the origin of chirality in bio(macro)molecules and thus in life [12];
3. the question why biomolecules on earth are based on carbon backbones and not on silicon;
4. the question whether one could imagine other forms of molecular architectures that make up self-repeating systems that develop under evolutionary conditions (molecular recognition, self-assembly and dynamic combinatorial chemistry) and how their molecular composition would be;
5. the question why nature developed DNA and RNA that utilize ribose and 2-deoxyribose as central nucleotide building blocks instead of the more abundant and readily available glucose [13].

For various reasons, however, falsifiability failed as a demarcation criterion. For example, it fails to describe how science actually works: Whether or not a particular hypothesis or theory is falsifiable is often far from straightforward, scientists often endorse non-falsifiable hypotheses and theories, and falsified hypotheses and theories often continue to be endorsed, as scientists have good reasons not to abandon them. Conversely, many claims that clearly do not belong into the scientific domain are falsifiable: consider explanations of events in everyday contexts. Since the failure of Popper’s attempt, philosophers of science have not been able to come up with a suitable alternative criterion and have largely given up on the project of distinguishing “good” science from other academic disciplines and from pseudo-science (but for a renewed interest in the issue, see [14]). It seems, then, that the question to focus on should not be what demarcates science from other areas of work.

An alternative approach considers the possible reduction of theories or entire sciences to more fundamental theories or sciences. While there are numerous models of reduction in the philosophy of science [15], a classic model is due to Ernest Nagel [16]. According to Nagel, the “reduction of one science to another” [16] involves connecting the concepts of both sciences by means of so-called bridge laws, and the derivation of the laws and theories of the reduced science from those of the more fundamental science. A well-known example is the reduction of thermodynamics to statistical mechanics [15,17], in which a bridge law would identify temperature with the mean kinetic energy of the constituent molecules of a gas. The science that is reduced is then explained by the science to which it is reduced, that is, the more fundamental science explains why the laws and theories of the less fundamental science hold [16]. As the “real” explanatory work is thus done at a more fundamental level, it might seem that the reduced science loses some of its scientific status. For chemistry this notion of reduction might have diverging implications. On the one hand, one might think that it should be possible to reduce many “molecularized” fields to chemistry, providing chemistry with the status of a comparatively fundamental field. On the other hand, one might think that chemistry could itself be reducible to a more fundamental science.

The question whether a particular field of work is science or should rather be seen as, for example, technology may thus be understood as the question whether the field has its own proper explanations, or accounts for phenomena by using explanations from other sciences located at a more fundamental level. On such an understanding, applied disciplines are not “really” explanatory, as their aim is not to explain the phenomena under study but rather to control them. Such a view can be found with philosopher of science Mario Bunge, who was among the first philosophers to address the question what makes a particular area of work into a science or a technological discipline, and what might be the relevant factors distinguishing science from technology. Bunge [18] distinguished between “pure” and “applied science”. According to Bunge, the distinction between the two has to do with the aims that are being pursued: “The method and the theories of science can be applied either to increasing our knowledge of the external and the internal reality or to enhancing our welfare and power. If the goal is purely cognitive, pure science is obtained; if primarily practical, applied science. […] [W]hereas the former wants to understand things better, the latter wishes to improve our mastery over them” [18]. An example of applied science in this sense would be cancer research, as it has a decidedly practical aim, namely curing cancer, rather than finding the laws of nature that govern cell growth.

With respect to organic chemistry, the relevant questions would thus be: Can the theories of organic chemistry be reduced to those of more fundamental sciences? Does organic chemistry have its own proper explanations? And does organic chemistry have a cognitive, explanatory aim, or rather a practical, applicatory aim (or perhaps both)? Note that answers to which extent organic chemistry does not have its own explanations, and has a practical rather than cognitive aim should not be thought of as involving a devaluation of the field. For Bunge, for example, the pure and applied sciences stood side by side as distinct modes of investigating the natural world. Both are knowledge-producing endeavors, but they produce different sorts of knowledge: knowledge about the world and knowledge of how the former type of knowledge can be applied to concrete problems, respectively [19]. Also, engineers and philosophers of technology have more recently pointed out that technological disciplines produce well-established knowledge and good explanations too, albeit knowledge and explanations of a different sort than is produced by the sciences [20–21].

The upshot of our discussion is simple: While at first glance the question whether a field such as organic chemistry can be counted as a science or as a different sort of endeavor, such as technology, might be considered trivial or pointless, much depends on how the question is conceived. Although an answer to the question will not change the field in practice, it will affect how organic chemists think of what they are doing: what it is their field aims for, how it goes about realizing those aims, and how it relates to other academic disciplines. While our aim is not to defend a particular answer to these questions, we want to suggest that achieving more clarity about such issues should be part and parcel of the professionalization of any academic. To further the discussion among the community of chemists, we have highlighted two ways these issues may be addressed.
